# Determination of Urinary Caffeine Metabolites as Biomarkers for Drug Metabolic Enzyme Activities

**DOI:** 10.3390/nu11081947

**Published:** 2019-08-19

**Authors:** Hyeong Jun Kim, Min Sun Choi, Shaheed Ur Rehman, Young Seok Ji, Jun Sang Yu, Katsunori Nakamura, Hye Hyun Yoo

**Affiliations:** 1 Institute of Pharmaceutical Science and Technology and College of Pharmacy, Hanyang University, Ansan-si, Gyeonggi-do 15588, Korea; 2 Department of Pharmacy, COMSATS Institute of Information Technology, Abbottabad 22060, Pakistan; 3 Hygiene House Healthcare Center (HHHC), Bannu 28100, Pakistan; 4 Department of Pharmacy, Ryukyu University Hospital, Okinawa 903-0215, Japan

**Keywords:** caffeine, metabolites, phenotyping, CYP450, NAT, xanthine oxidase

## Abstract

Caffeine is commonly taken via the daily dietary consumption of caffeine-containing foods. The absorbed caffeine is metabolized to yield various metabolites by drug-metabolizing enzymes, and measuring the levels of each caffeine metabolite can provide useful information for evaluating the phenotypes of those enzymes. In this study, the urinary concentrations of caffeine and its 13 metabolites were determined, and the phenotypes of drug metabolic enzymes were investigated based on the caffeine metabolite ratios. Human urine samples were pretreated using solid phase extraction, and caffeine and its metabolites were analyzed using liquid chromatography-tandem mass spectrometry. Based on the urinary caffeine metabolite concentrations, the caffeine metabolite ratios were calculated for six human subjects at specified time points after caffeine intake. Variations in urinary metabolite levels among individuals and time points were reported. In addition, the resultant enzyme activities showed different patterns, depending on the metabolite ratio equations applied. However, some data presented a constant metabolite ratio range, irrespective of time points, even at pre-dose. This suggests the possibility of urinary caffeine metabolite analysis for routine clinical examination. These findings show that urinary caffeine and the metabolite analysis would be useful in evaluating metabolic phenotypes for personalized medicine.

## 1. Introduction

Caffeine, an alkaloid of the methylxanthine class, is the world’s most widely consumed psychoactive substance. As a naturally occurring substance, caffeine is found in the leaves, fruits, or seeds of more than 60 plant species. Caffeine is popularly and extensively taken via the daily dietary consumption of caffeine-containing beverages or foods [1,2].

**Figure 1:**
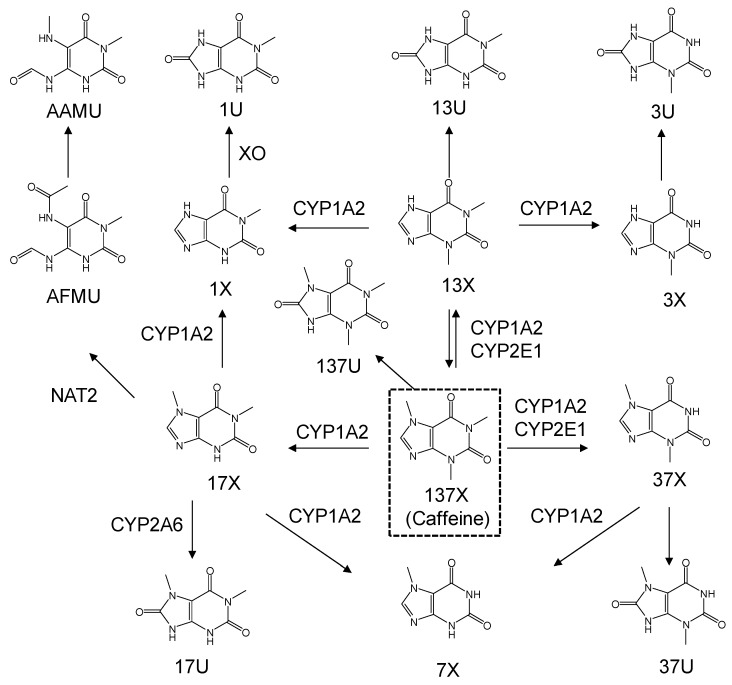
The metabolic pathway of caffeine.

In the liver, caffeine is subjected to a series of metabolic reactions to yield a mixture of N-methylated xanthines, uric acids, and an acetylated uracil, as its metabolites [3]. There are various metabolic enzymes involved in each caffeine metabolic pathway (Figure 1). These enzymes include N-acetyltransferase 2 (NAT2), xanthine oxidase (XO), and cytochrome P450—particularly 1A2 (CYP1A2) and 2A6 (CYP2A6)—which are of prime interest and must be phenotypically evaluated because of their roles in metabolizing various xenobiotics [4,5,6]. These four enzymes involved in caffeine metabolism display genetic polymorphism, and their metabolizing activities can vary in individuals [4,5,6]. Accordingly, inter-individual variability can be observed in caffeine and its metabolite levels, or their ratios in biological fluids or tissues. In this context, measuring the levels of caffeine and each caffeine metabolite can provide useful information for evaluating the phenotypes of drug-metabolizing enzymes. Furthermore, caffeine is popularly, and even routinely, consumed worldwide as various types of foods, such that caffeine or its metabolites are likely to be detected in urine. Due to these aspects, the measurement of urinary caffeine metabolite levels can be an advantageous marker for the phenotyping of individual drug-metabolizing activities.

Several analytical methods have been reported for measuring caffeine and its metabolites in urine using high-performance liquid chromatography (HPLC) or high-performance liquid chromatography-tandem mass spectrometry (LC-MS/MS) [7,8,9,10,11,12,13,14,15,16]. Based on such analytical methods, the phenotyping of CYP1A2, CYP2A6, NAT2, or XO enzyme activity has been investigated by measuring urinary caffeine and its metabolites in subjects receiving a regulated dietary caffeine intake and in uncontrolled subjects [7,9,10,12,13,17,18,19,20]. However, each study evaluated the enzyme phenotypes based on different metabolite ratio equations for a limited population, and information on the feasibility of those methods is still insufficient for general, practical application.

In this study, the urinary concentrations of caffeine and its metabolites were determined using LC-MS/MS analysis. The resulting concentration data was applied to various caffeine metabolite ratio equations to determine the phenotypes of each drug metabolic enzyme. The feasibility of phenotyping the drug-metabolizing enzyme based on urinary caffeine metabolite ratios was examined.

## 2. Materials and Methods

### 2.1. Chemicals and Reagents

Chemicals including, 1-methylxanthine (1X), 3-methylxanthine (3X), 7-methylxanthine (7X), 1,3-dimethylxanthine (theophylline, 13X), 1,7-dimethylxanthine (paraxanthine, 17X), 3,7-dimethylxanthine (theobromine, 37X), 1,3,7-trimethylxanthine (caffeine, 137X), 1-methyluric acid (1U), 1,3-dimethyluric acid (13U), 1,7-dimethyluric acid (17U), 3,7-dimethyluric acid (37U), and 1,3,7-trimethyluric acid (137U), and acetic acid were provided by Sigma-Aldrich (St. Louis, MO, USA). The following chemicals were procured from Santa Cruz (Dallas, TX, USA): 5-acetylamino-6-amino-3-methyluracil (AAMU), 5-acetylamino-6-formylamino-3-methyluracil (AFMU) and internal standards (IS) including 1-methylxanthine-2,4,5,6-13C4 (1X*), 1,3,9-15N3, and 1-methyluricacid-2,4,5,6-13C4,1,3,9-15N3 (1U*). HPLC-grade acetonitrile was purchased from J. T. Baker (Philipsburg, NJ, USA). Water was prepared using a Milli-Q purification system (Millipore, Bedford, MA, USA). All other chemicals used were of analytical grade and used as received. All the standard solutions and mobile phases were passed through a 0.22-µm membrane filter before use.

### 2.2. Human Urine Specimens

The study protocol and consent forms were approved by the Institutional Review Board of the Hanyang University, and all the participants provided written informed consent to participate in the study. The eligibility criteria for the study included physically healthy ethnic Korean adult men (19 years of age or older) who signed written informed consent. Participants were excluded if they were being treated for acute disease or other diseases or who needed treatment, or were receiving any medication that might affect the metabolism or excretion of caffeine. Urine samples were collected from 6 volunteers prior to the consumption of a caffeine-containing drink (120 mg of caffeine intake), and 1 h, 2 h, 4 h, 6 h, 8 h, and 10 h after the drink. Blank urine samples were obtained from healthy volunteers who had not consumed any methyl xanthine-containing food or beverage for the last 24 h. The urine samples were collected in clear 15-mL centrifuge tubes. All the study procedures were conducted in compliance with the principles of Declaration of Helsinki and Korean Good Clinical Practice guidelines (IRB HYG-16-193-2).

### 2.3. Urine Sample Preparation and Standard Samples

For the LC–MS/MS analysis, 100 µL of urine was added to 10 mL of 0.1% acetic acid with IS. Then, 1 mL of diluted mixture was passed through pre-activated Sep-Pak C18 cartridges (96-well type OASIS HLB extraction cartridge, Waters). The cartridge was washed with 1 mL of 0.1% formic acid two times, and then eluted with 1 mL of methanol. The eluate was dried under nitrogen gas. The residue was resolved in 0.1% acetic acid/acetonitrile (90:10, 100 µL), and a 5-µL aliquot was injected into the HPLC column for LC-MS/MS analysis. The analyte mixture was dissolved in MeOH at a concentration of 1 mg/mL and diluted to a series of working standard solutions. A 5-µL aliquot of each working standard solution was spiked to 95 µL of human blank urine. Then, the spiked samples were pretreated as described above. The concentrations of QC samples for each analyte are provided as supplementary data (Table S1).

### 2.4. Method Validation

The developed method was validated according to the US Food and Drug Administration (FDA) guidelines as mentioned in the “Guidance for Industry, Bioanalytical Method Validation, 2018” [21].

#### 2.4.1. Selectivity, Linearity, and LLOQ

The selectivity of the method was assessed by comparing multiple reaction monitoring (MRM) chromatograms between a blank sample and a standard spiked mixture. Lower limits of quantitation (LLOQs) for each analyte were determined considering the concentration level found in human urine samples, and evaluated for accuracy and precision. The calibration curves were prepared using the samples at concentration ranges depending on their LLOQ. The calibration curves were generated by plotting the peak area ratios of the analytes/IS versus the concentrations in the standard spiked samples. The linear correlation coefficient (r2) for all the calibration curves should be greater than 0.99.

#### 2.4.2. Precision and Accuracy

To assess the intra-day precision and accuracy, QC samples were analyzed, in triplicate, at different concentration levels (*n* = 3) on the same day. In case of inter-day assays, the precision and accuracy were assessed by determining the QC samples over three consecutive days. The accuracy was measured as a deviation of the calculated mean value from the nominal mean value, which should be within 15% of the nominal value except for LLOQ, which should not exceed 20% of the nominal value. The precision was determined at each concentration level, in terms of percent relative standard deviation (%RSD), which should not exceed 15% of the nominal concentration, except for the LLOQ, where it should not deviate by more than 20%.

#### 2.4.3. Matrix Effect and Recovery

The matrix effect was evaluated by comparing the spiked QC samples at low, middle, and high concentrations in the blank, to the same QC sample in 0.1% acetic acid. The recovery was determined by comparing the reaction of the extracted sample, to which the analyte is added, and the biological sample after extraction.

#### 2.4.4. Stability

Stability was evaluated for the QC samples under various conditions such as freeze-and-thaw, short-term, long-term, and processed sample stability. For the freeze-and-thaw stability test, three aliquots of the QC samples were stored at −20 °C for 24 h and thawed at room temperature. When completely thawed, the samples were refrozen for 24 h under the same condition, and this was repeated three times. For short-term stability, the QC samples were maintained at room temperature for 12 h, and then analyzed. For long-term stability, the QC samples were stored at −20 °C for 7 days, and then analyzed. The post-preparative stability was evaluated by analyzing the QC samples placed in the autosampler for 24 h at 4 °C.

### 2.5. LC-MS/MS Analysis

The LC-MS/MS system consisted of a Shiseido SP LC SP3202 binary pump HPLC system (Tokyo, Japan) and TSQ Quantum™ Access MAX Triple Quadrupole Mass Spectrometer (Thermo Fisher Scientific, Waltham, MA, USA), equipped with an electrospray ionization (ESI) source. Chromatographic separation was achieved on a Kinetex C18 column (3.0 × 100 mm, 2.6 µm; Phenomenex, Torrance, CA, USA) at a temperature of 40 °C. The HPLC mobile phases consisted of two solvents: (A) 0.1% acetic acid and (B) acetonitrile in 0.1% acetic acid. A linear gradient program was used with a flow rate of 0.2 mL/min. The initial mobile phase was set at 15% of solvent B and gradually increased to 90% in 3 min, kept at 90% for 1 min, and then followed by re-equilibrium for 3 min. Electrospray ionization (ESI) was performed in both positive and negative ion mode, with nitrogen as the nebulizing agent, spray voltage, sheath gas pressure, and aux gas pressure at optimal values of 3000, 60, and 20 (arbitrary units), respectively. The capillary temperature was 350 °C. Multiple reaction monitoring (MRM) detection was employed. The precursor–product ion pairs used in MRM mode are provided as supplementary data (Table S2).

### 2.6. Metabolic Ratio Calculation

The urinary caffeine and its metabolite concentrations were measured using the LC-MS/MS analysis. The resulting data were evaluated using the equations for the metabolic ratio calculation. The molar urinary ratios specific for each drug-metabolizing enzyme were calculated referring to the equations previously reported [4,10,17,18,20]. Thus, a higher metabolic ratio indicates a higher enzyme activity.

## 3. Results

### 3.1. LC-MS/MS

Caffeine, 1X, 7X, 17X, 37X, 13U, 17U, 37U, 137U, AAMU, and 1X* were ionized to yield the protonated molecular ions ([M+H]^+^) at m/z 195.2, 167.1, 167.0, 181.2, 181.2, 197.2, 197.1, 197.1, 211.2, 199.2, and 174.1, respectively. Additionally, 3X, 13X, 1U, AFMU, and 1U* were ionized to yield the deprotonated molecular ions ([M−H]^−^) at m/z 164.9, 179.1, 181.1, 225.1, and 188.1, respectively. Ion polarity switching was applied for the simultaneous detection of protonated and deprotonated ions. Water and acetonitrile were used as the mobile phase solutions; to increase the response of 1U, 0.1% acetic acid was added to both mobile phase solvents. Using the gradient elution, all the analytes were eluted within 5 min. The representative LC-MS/MS extracted ion chromatograms are provided in the supplementary data (Figure S1).

### 3.2. Method Validation

The calibration curves for each analyte were linear over each corresponding, selected concentration range, with correlation coefficient (r2) values greater than 0.99. The linear ranges for caffeine and its metabolites are presented in the supplementary data (Table S2). The LLOQ values for all analytes ranged from 10 ng/mL to 166 ng/mL with an accuracy of approximately 91.4% to 114.0% and a precision of ≤16.3%.

The intra-day precision was less than 16.4%, while the accuracy (as a percentage of relative error values) was within the range of ±11.9% at the tested QC concentrations. The inter-day assay also showed satisfactory accuracy and reproducibility, with a precision of less than 11.4%, and an accuracy within the range of ±14.0% at the tested QC levels. These results are summarized in Table 1.

The matrix effect was negligible for caffeine and its metabolites, except for 1X, 137U, and AAMU, which seemed to be affected by it. However, they showed acceptable RSD criteria (within ±15%). For recovery evaluation, caffeine and all its metabolites were stable and well recovered (%RSD, <10.6) from samples. The matrix effect and recovery data are provided in the supplementary data (Tables S3 and S4).

In all the tested conditions, caffeine and its metabolites were shown to be stable, with acceptable recovery (RSD within ±15%), except for the long-term stability of AAMU and AFMU (Table S5). The accuracy and RSD values were within ±12.4% and ±9.4% for freeze-and-thaw stability, within ±11.6% and ±9.7% for short-term stability, within ±12.3% and ±10.8% for long-term stability, and within ±12.1% and ±8.2% for processed sample stability. Meanwhile, the long-term stability of AAMU was 323.4%, and that of AFMU was 47.2%. AFMU is known to be spontaneously converted to AAMU, and the present results may reflect this phenomenon. According to Nyeki et al. [22], the conversion of AFMU into AAMU is not only subjected to nonenzymatic hydrolysis in urine, but is also NAT2 phenotype-dependent. Nevertheless, it would be better to analyze urine samples immediately after voiding to minimize errors in calculating the metabolite ratio for enzyme phenotyping.

### 3.3. Enzyme Phenotyping Based on Urinary Caffeine Metabolite Ratio

**Figure 2:**
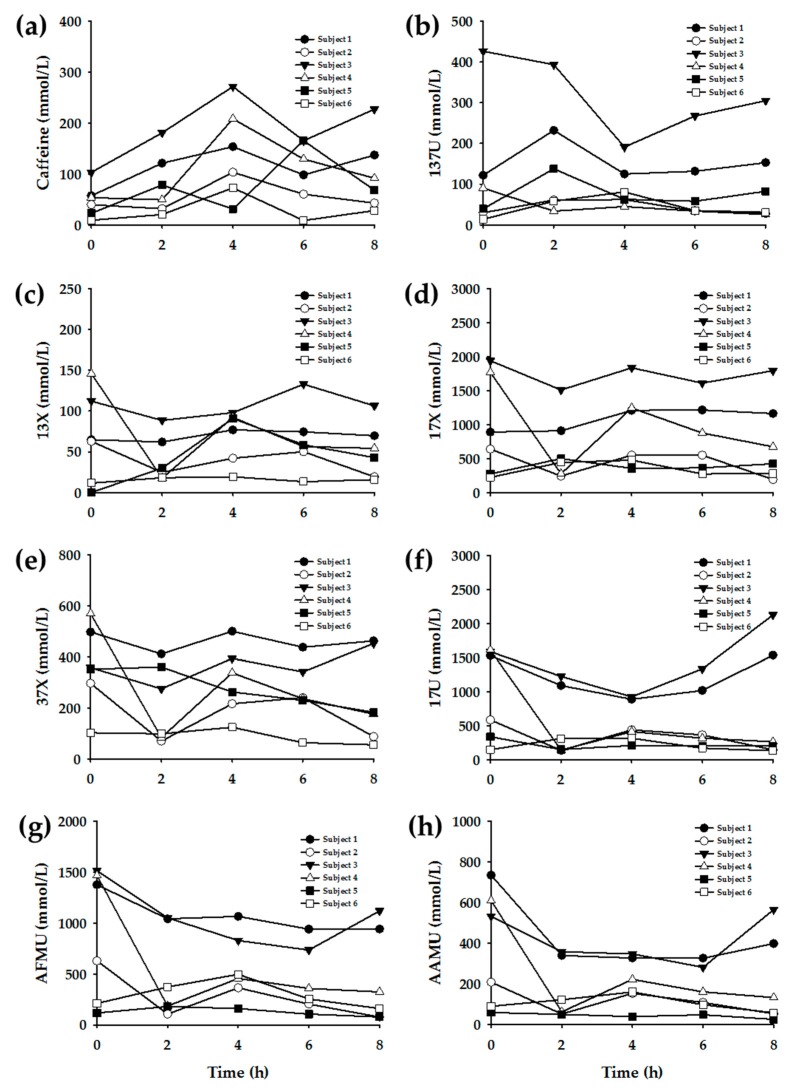

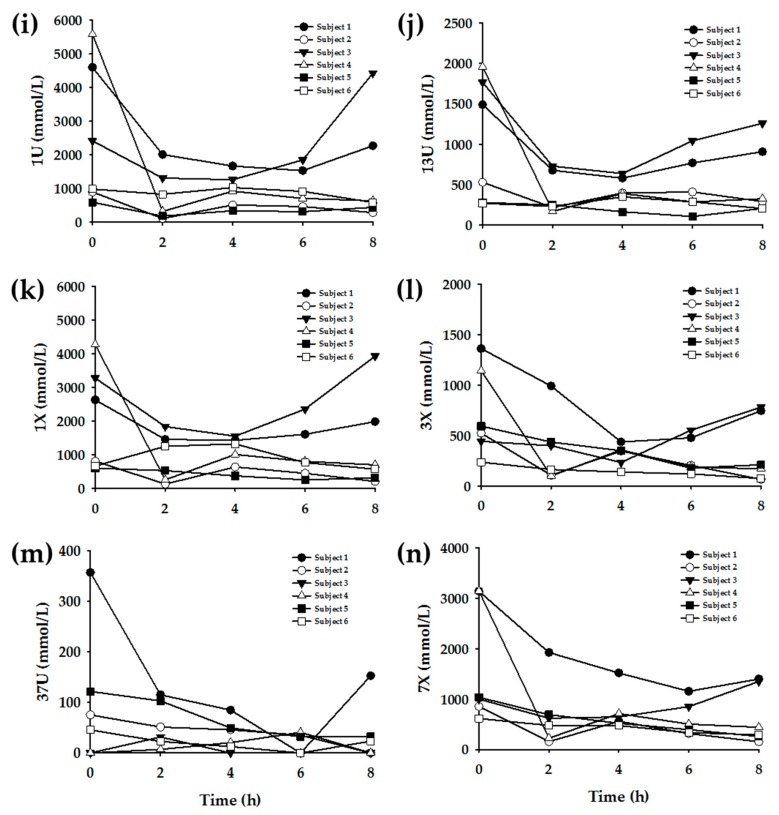
Urinary concentration levels of caffeine and its metabolites in six subjects. (**a**) caffeine, (**b**) 1,3,7-trimethyluric acid (137U), (**c**) 1,3-dimethylxanthine (13X), (**d**) 1,7-dimethylxanthine (17X), (**e**) 3,7-dimethylxanthine (37X), (**f**) 1,7-dimethyluric acid (17U), (**g**) 5-acetylamino-6-formylamino-3-methyluracil (AFMU), (**h**) 5-acetylamino-6-amino-3-methyluracil (AAMU), (**i**) 1-methyluric acid (1U), (**j**) 1,3-dimethyluric acid (13U), (**k**) 1-methylxanthine (1X), (**l**) 3-methylxanthine (3X), (**m**) 3,7-dimethyluric acid (37U), and (**n**) 7X.

The urinary caffeine metabolite levels in the six subjects are as shown in Figure 2. When the six urine samples were analyzed, the targeted caffeine metabolites were successfully detected in most of the samples. The urinary concentration ranges of each metabolite were tabulated in Table 2. The measured metabolite concentrations exhibited large variations among individuals and time points. However, the concentration ranges were generally consistent with previously reported values [8].

**Figure 3:**
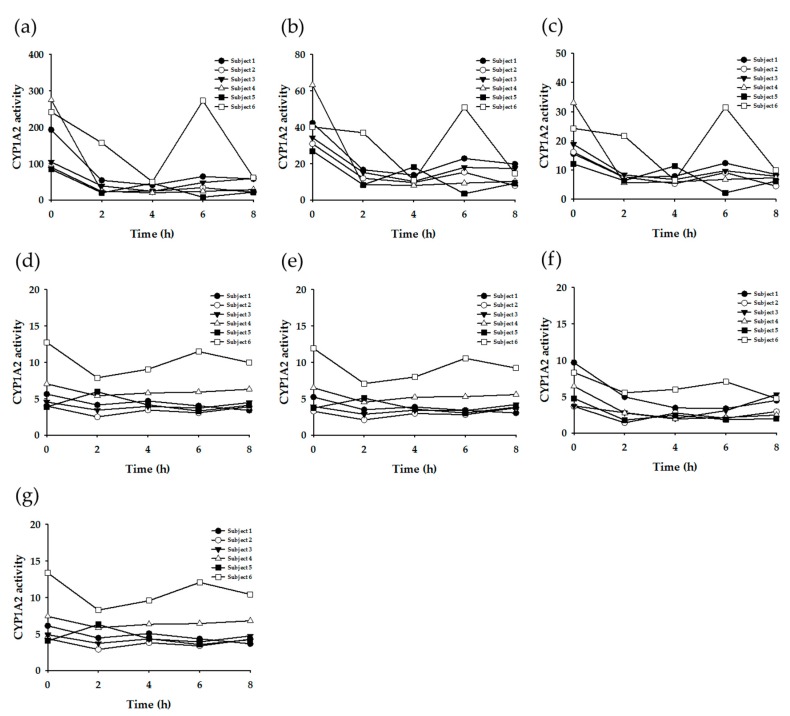
Plots of metabolite ratio for CYP1A2. The metabolic ratio equations used are as follows: (**a**) (AFMU + 1X + 1U + 17X + 17U)/137X, (**b**) (17X + 17U)/137X, (**c**) 17X/137X, (**d**) (AAMU + 1X + 1U)/17U, (**e**) (AFMU + 1X + 1U)/17U, (**f**) (AFMU+1X+1U)/17X, (**g**) (AAMU+AFMU+1X+1U)/17U.

**Figure 4:**
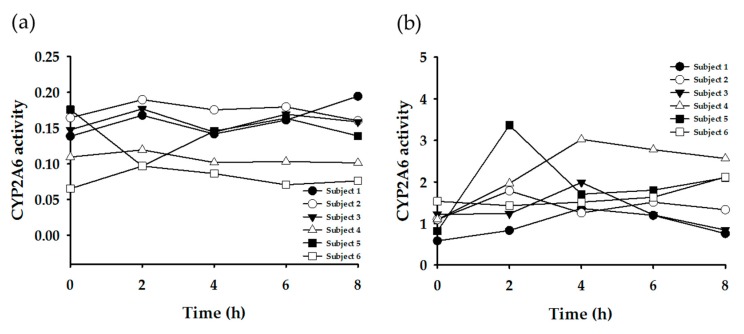
Plots of metabolite ratio for CYP2A6. The metabolic ratio equations used are as follows: (**a**) 17U/(AFMU + 1U + 1X + 17X + 17U), (**b**) 17X/17U.

**Figure 5:**
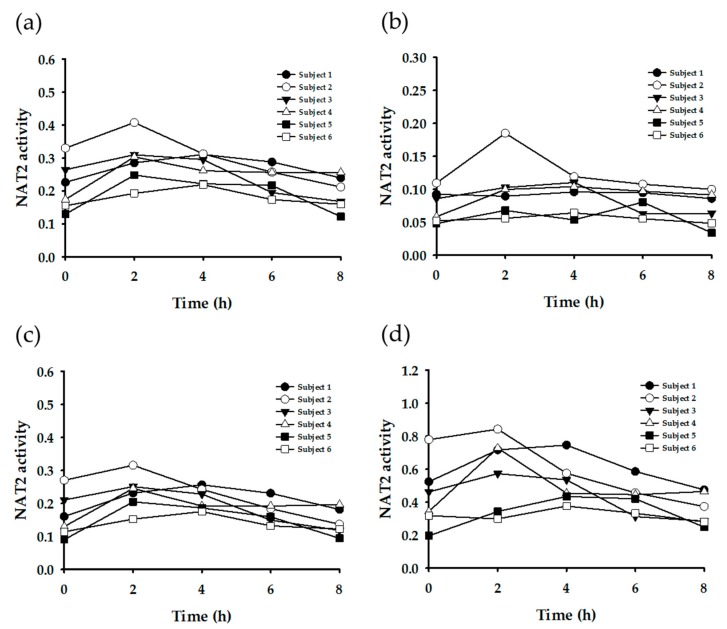
Plots of metabolite ratio for NAT2. The metabolic ratio equations used are as follows: (**a**) (AAMU+AFMU)/(AAMU+AFMU+1X+1U), (**b**) AAMU/(AAMU+1X+1U), (**c**) AFMU/(AFMU+1X+1U), (**d**) AFMU/1X.

**Figure 6:**
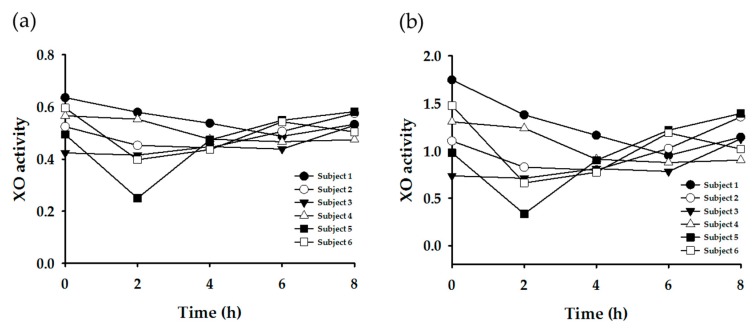
Plots of metabolite ratio for XO. The metabolic ratio equations used are as follows: (**a**) 1U/1X + 1U, (**b**) 1U/1X.

Subsequently, enzyme-specific metabolite ratios were calculated based on the urinary concentration data. Referring to the extant literature, the urinary metabolite concentrations were applied to various equations to yield enzyme-specific metabolite ratios (Table 3). Figure 3, Figure 4, Figure 5 and Figure 6 display the plots for each enzyme activity in individuals, which is expressed as the metabolite ratio. The metabolite ratio patterns varied between individuals and time points, depending on the equations applied, even for identical enzymes.

To investigate the CYP1A2 phenotypes, seven equations were tested. The resulting metabolic ratio plots are shown in Figure 3. Figure 3a–c exhibited a considerable difference between pre-dose (0 h) and post-dose data. The metabolic ratio patterns of Subject #6 were generally different from those of other subjects.

To investigate the CYP2A6 phenotypes, two equations were tested (Figure 4). Figure 4a generally showed constant metabolite ratio patterns within individuals (except for Subject #5) over all of the time points. However, Figure 4b showed a larger variation according to time. The order of the metabolite ratio values for all six subjects (i.e., the relative metabolic activity) was not consistent between two plots.

NAT2 activity was tested with four different equations. Three equations (Figure 5a–c) generated similar metabolite ratio patterns, whereas the other (Figure 5d) showed a large variation between individuals and time points.

The XO activity was evaluated using two equations. The metabolite ratio plots are shown in Figure 6. The resultant patterns were generally similar between the two plots, but the variation was larger in plot (b).

## 4. Discussion

This study measured the urinary concentrations of caffeine and its 13 metabolites for 8 h, including a pre-dose time point, using LC-MS/MS. The metabolic ratios for phenotyping the drug metabolizing enzymes were calculated based on the various equations previously reported. Subsequently, the resulting metabolic ratios or patterns were compared, and their validity and feasibility were investigated.

The resulting urinary caffeine concentration data showed a relatively obvious increase and decrease pattern across time, which indicated the absorption and elimination of caffeine after oral intake. Meanwhile, the changes in caffeine metabolite concentration levels, according to time points, were not as evident as those of caffeine. However, caffeine metabolites also showed a weak pattern of slow increase after caffeine intake, on excluding the data at pre-dose. Most metabolites showed the highest concentrations at pre-dose, which was presumably due to the urine concentration. Thus, the urine was diluted in post-dose samples, as the urine was frequently collected (i.e., at 2-h intervals) after caffeine intake; meanwhile, at pre-dose, the caffeine metabolites, which resulted from usual dietary caffeine intake, could be detected at higher concentrations in relatively concentrated urine.

The most diverse equations have been suggested to determine the enzyme activity of CYP1A2 in previous research. This study applied seven equations to yield the metabolite ratio. Among them, three equations (Figure 3a–c) showed a significant difference between pre-dose (0 h) and post-dose data, and the within-individual variation was large according to the time points. Meanwhile, the other four plots showed a more constant ratio pattern within individuals. The caffeine (137X) concentration was included in the former three equations (Table 3: Equations CYP1A2-(a), (b), and (c)). Accordingly, the activity showed a large difference in the results between pre-dose and post-dose. However, the other equations showed relatively constant results over time, even between pre-dose and post-dose measurements, as those equations did not involve caffeine concentration. Generally, the CYP1A2 activity of Subject #6 appeared to be higher than that of the other subjects, but plot F (Figure 2f) did not exhibit this tendency. These findings suggest that Equations CYP1A2-(d), (e), and (g) (Table 3) may be more appropriate for determining the CYP1A2 phenotype.

Acetylation is a primary route for the biotransformation of many hydrazine drugs, which is mediated by NAT [4]. The polymorphism of NAT (in particular, NAT2) is responsible for the inter-individual variability in the acetylation of drugs. Thus, the population can be categorized into rapid acetylators and slow acetylators, according to their NAT2 phenotypes [6]. Such acetylation polymorphism is reported to vary among ethnic groups [6]. It is known that rapid acetylators are dominant in the Korean population [23]. In the present study, when the metabolite ratios were calculated by the equation (AFMU + AAMU)/(AFMU + AAMU + 1X + 1U) (corresponding to Figure 4a) and evaluated by the criteria reported by Jetter et al. [10,20], the six subjects tested in this study were determined to be rapid acetylators. This seems to be reasonable based on the generally recognized facts on acetylation polymorphism in Koreans. However, when evaluated by the criteria based on other equations, such as AFMU/(AFMU + 1U + 1X) or AAMU/(AAMU + 1U + 1X) [7,13,24], these six subjects were shown to be slow acetylators. Therefore, to determine the phenotypes exactly, comprehensive genotyping data is necessary.

Meanwhile, the time point for sample collection may affect the metabolic ratio results, as the rate of metabolic reaction may be different depending on each metabolic pathway. Jetter et al. (2009) reported that the timing of urine collection can affect XO phenotyping results [20]. However, this study’s data, generated from the same equation [1U/(1U + 1X)] (Figure 5a), did not show a significant variation according to the urine collection time, except in Subject #5. This suggests the possibility that spot urine samples, under normal dietary conditions, can be used for XO phenotyping.

Recently, research on polymorphism and personalized medicine has been extensive. Genotyping and phenotyping for drug metabolizing enzymes are vital strategies for characterizing the polymorphism of drug-metabolizing enzymes in individuals for personalized medicine. However, evaluating the phenotype is more critical than evaluating the genotype in some enzymes. For example, XO is a form of xanthine oxidoreductase, which is a type of enzyme that generates reactive oxygen species [25]. These enzymes catalyze the oxidation of hypoxanthine to xanthine and can further catalyze the oxidation of xanthine to uric acid. Xanthine oxidase plays a crucial role in many drug metabolic processes, such as thiopurine drugs, containing 6-mercaptopurine, allopurinol, and uric acid, etc. [26,27,28,29]. Xanthine oxidase is important in gout patients, because XO produces uric acid, which is a crucial factor in gout. In addition, xanthine oxidase is involved in the catabolism of xenobiotics; for example, it converts a prodrug (mercaptopurine) into the active form 6-thioinosine-5′triphosphate [30]. About 20 genetic variants are reported, and each XO variant may differ in its enzymatic activity [20]; however, decreased enzyme activity is shown only in 4% or fewer volunteers [31]. Thus, it would be difficult to explain the cause of the variations in XO activity on the basis of genetic polymorphisms alone [20]. Therefore, it is meaningful to establish an optimized method for assessing the activity of the drug-metabolizing enzymes, including XO, for phenotyping.

Meanwhile, it has been recognized that differences in the activity of enzymes involved in nicotine metabolism are partly responsible for inter-individual variation in lung cancer risk among smokers [32,33,34,35]. CYP2A6 is a principal enzyme in nicotine metabolism, and CYP2A6-mediated C-oxidase activity has been reported to correlate with exposure to carcinogens by smoking [32,33,34,35]. Many reports have demonstrated that CYP2A6 variants that exert reduced enzymatic activity are associated with lower lung cancer risk [32,33,34,35]. However, these genetic polymorphisms are reported to account for only a portion of the variation in CYP2A6 activity [4]. Therefore, the phenotyping of CYP2A6 activity could be more appropriate for estimating lung cancer risk related to nicotine metabolism. In this context, the urinary caffeine metabolite ratio is a useful biomarker for predicting the risk of lung cancer.

The limitations of this study are its small sample size, the lack of genotyping data, and that this data was obtained from a single set of experiments. Nevertheless, this study enables the evaluation of phenotyping results by demonstrating the caffeine metabolite ratio plots generated from different phenotyping equations. In addition, the data obtained at different time points, including the pre-dose and post-dose suggests the possibility that the enzyme phenotyping for CYP1A2, CYP2A6, NAT2, and XO can be conducted as a routine urine test, without the administration of drugs.

## 5. Conclusions

Caffeine is popularly present in a wide variety of foods and beverages, and is extensively consumed via the daily diet. By measuring the exposure to caffeine in biological samples, such as the urine, the extent of caffeine consumption could be directly indicated. However, caffeine can be also used as a biomarker to indicate the activities of drug-metabolizing enzymes. This study demonstrated the possibility that enzyme phenotyping based on urinary caffeine metabolite ratios can be routinely used under general dietary conditions. This suggests a possibility for urinary caffeine metabolite analysis as a routine clinical examination. Urinary caffeine and its metabolite analysis would be useful in evaluating drug metabolic phenotypes for personalized medicine.

## Figures and Tables

**Table 1:** Intra-day and inter-day accuracy (A) and precision for the determination of caffeine and its metabolites

	Intra-Day Assay (*n* = 5)								Inter-Day Assay (*n* = 5)							
Analyte	LLOQ		Low QC		Middle QC		High QC		LLOQ		Low QC		Middle QC		High QC	
	A	RSD	A	RSD	A	RSD	A	RSD	A	RSD	A	RSD	A	RSD	A	RSD
	(%)	(%)	(%)	(%)	(%)	(%)	(%)	(%)	(%)	(%)	(%)	(%)	(%)	(%)	(%)	(%)
137U	106.3	3.0	115.0	1.1	104.2	1.1	108.7	0.9	108.6	8.1	113.0	3.5	104.7	5.9	106.3	1.9
13X	102.4	16.3	94.0	4.9	100.4	5.2	101.5	4.1	91.4	10.8	91.2	4.5	94.3	6.7	96.0	11.0
17X	109.0	3.3	112.4	2.4	101.8	2.5	101.1	9.6	109.4	3.0	110.0	3.8	108.6	4.0	100.4	1.7
37X	102.5	4.6	105.2	3.8	98.2	3.6	99.4	5.2	106.5	7.4	100.8	4.1	104.9	3.3	101.5	2.6
17U	102.6	2.0	102.9	9.9	86.6	1.6	95.7	4.7	106.4	11.4	90.5	6.8	87.0	2.0	95.0	3.3
AFMU	103.4	8.1	96.8	4.8	98.9	1.8	89.1	5.5	95.0	5.6	92.5	3.9	95.6	4.0	89.1	2.2
AAMU	111.9	7.7	107.4	5.6	105.3	4.5	101.0	7.0	106.0	6.8	106.6	5.6	103.0	2.7	107.4	4.1
1U	104.3	3.3	101.0	1.9	102.8	7.3	88.7	2.4	114.0	6.3	105.2	6.8	107.5	7.8	88.1	1.8
13U	104.7	11.4	96.4	9.7	113.3	1.1	108.0	2.3	110.9	4.7	99.3	9.3	114.3	0.5	111.7	2.6
1X	104.4	6.0	108.7	5.8	101.9	1.3	102.2	7.2	106.0	7.0	100.7	10.3	101.0	3.7	96.8	6.2
3X	101.7	12.9	101.5	3.9	102.4	9.1	104.3	5.9	97.0	2.9	97.2	9.4	98.2	7.8	107.7	4.3
37U	96.3	6.1	97.1	5.1	102.7	11.7	101.9	1.7	102.4	4.2	100.0	6.3	97.4	8.3	100.0	5.5
7X	108.6	2.1	103.4	7.7	98.7	2.0	101.0	7.2	103.3	6.3	106.1	3.7	100.2	9.6	97.2	6.3
137X	92.8	8.7	107.3	3.9	103.7	9.9	103.5	6.6	93.4	7.4	108.9	8.4	110.1	3.0	105.5	9.5

LLOQ: lower limits of quantitation, %RSD: percent relative standard deviation. QC: quality control.

**Table 2:** Urinary concentration ranges of caffeine and its metabolites.

Metabolite	Concentration Range (μM)	Metabolite	Concentration Range (μM)
137U	13.9–426.3	1U	104.5–5577.5
13X	0–145.9	13U	104.6–1957.9
17X	191.8–1941.3	1X	126.3–4273.4
37X	54.9–569.3	3X	65.2–1362.3
17U	131.6–2127.2	37U	0–357.2
AFMU	76.8–1514.9	7X	154.3–3145.5
AAMU	26.1–735.0	137X (caffeine)	8.7–271.7

**Table 3:** Equations of caffeine metabolic ratios used for enzyme-specific activities.

Enzyme		Equation	Reference
CYP1A2	(a)	(AFMU + 1X + 1U + 17X + 17U)/137X	[4,17,18]
	(b)	(17X + 17U)/137X	[4]
	(c)	17X/137X	[4]
	(d)	(AAMU + 1X + 1U)/17U	[4]
	(e)	(AFMU + 1X + 1U)/17U	[4]
	(f)	(AFMU + 1X + 1U)/17X	[4]
	(g)	(AAMU + AFMU + 1X + 1U)/17U	[4]
CYP2A6	(a)	17U/(AFMU + 1U + 1X + 17X + 17U)	[4]
	(b)	17X/17U	[4]
NAT2	(a)	(AAMU + AFMU)/(AAMU + AFMU + 1X + 1U)	[4,10,20]
	(b)	AAMU/(AAMU + 1X + 1U)	[4]
	(c)	AFMU/(AFMU + 1X + 1U)	[4]
	(d)	AFMU/1X	[4]
XO	(a)	1U/1X + 1U	[4,20]
	(b)	1U/1X	[4]

## Supplementary Materials

The following are available online at https://www.mdpi.com/2072-6643/11/8/1947/s1, Figure S1: Representative LC-MS/MS extracted ion chromatograms for caffeine and its metabolites in human urine at 2 h after administration of caffeine-containing drink, Table S1: Concentrations of quality control standards for caffeine and its metabolites, Table S2: Calibration range, retention time and multiple reactions monitoring data for caffeine and its metabolites, Table S3: Matrix effect data for caffeine and its metabolites, Table S4: Recovery data for caffeine and its metabolites, Table S5: Stability of caffeine and its metabolites in various conditions.
