# Supplementary material for: Determination of Urinary Caffeine Metabolites as Biomarkers for Drug Metabolic Enzyme Activities
Source: Nutrients. 2019 Aug 19;11(8):1947. doi: 10.3390/nu11081947 (PMC6723199; doi:10.3390/nu11081947)
Supplement: Supplementary file 1 [file nutrients-11-01947-s001.pdf]

Supplementary data

Determination of urinary caffeine metabolites as biomarkers for drug metabolic enzyme activities

Hyeong Jun Kim, Min Sun Choi, Shaheed Ur Rehman, Young Seok Ji, Jun Sang Yu, Katsunori Nakamura, Hye Hyun Yoo[a,*]

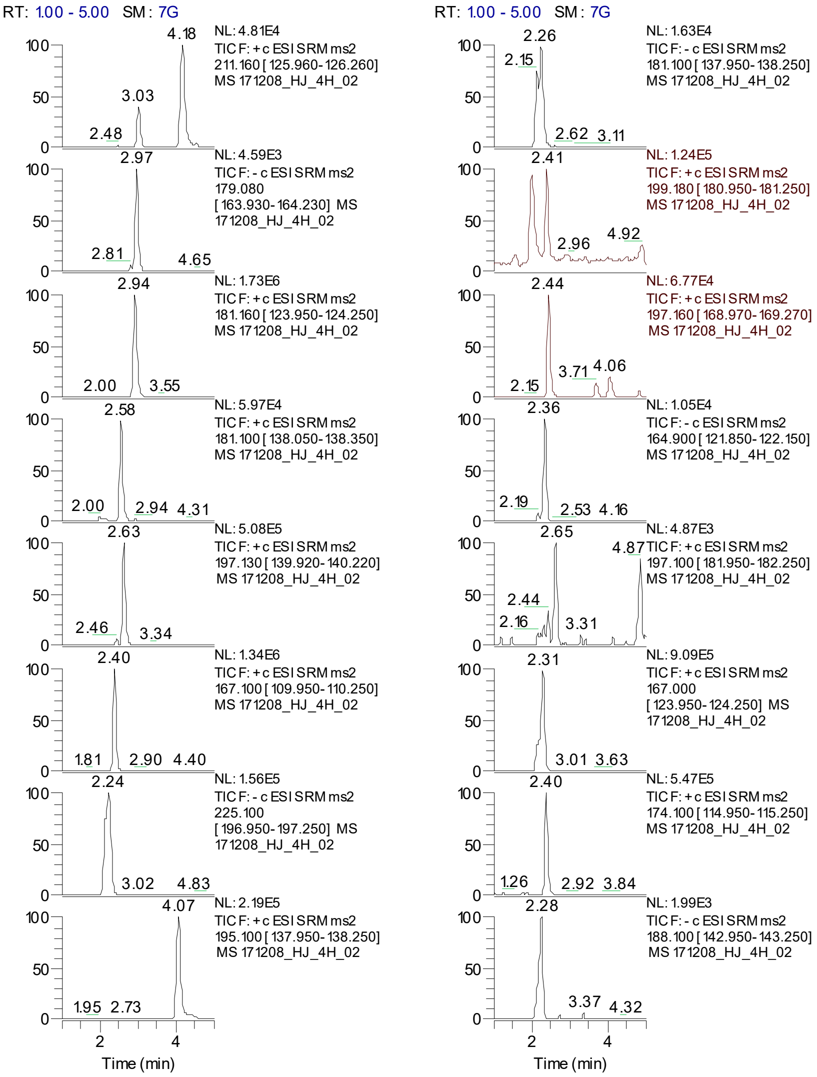

Figure S1. Representative LC-MS/MS extracted ion chromatograms for caffeine and its metabolites in human urine at 2 h after administration of caffeine-containing drink

Supplementary data

Table S1. Concentrations of quality control standards for caffeine and its metabolites

Analyte	Concentration (ng/mL)			
	**LLOQ**	**LOQ**	**MLQ**	**HLQ**
Caffeine	13	32	320	1600
137U	10	24	120	400
13X	30	60	240	800
17X	50	120	1200	6000
37X	100	240	1200	4000
17U	67	160	1600	8000
AFMU	133	320	3200	16000
AAMU	33	48	240	800
1U	150	300	1200	4000
13U	30	75	360	1200
1X	166	400	4000	20000
3X	100	240	1200	4000
37U	30	60	240	800
7X	50	120	1200	6000

Supplementary data

Table S2. Calibration range, retention time and multiple reactions monitoring data for caffeine and its metabolites

Analyte	Calibration range (ng/mL)	RT (min)	Ion mode	Precursor ion (m/z)	Product ion (m/z)
Caffeine	13-2000	4.00	+	195.20	138.10
137U	10-500	3.06	+	211.16	126.10
13X	30-1000	2.99	-	179.08	164.08
17X	50-7500	2.96	+	181.16	124.10
37X	100-5000	2.59	+	181.17	138.20
17U	67-10000	2.66	+	197.13	140.07
AFMU	133-20000	2.37	-	225.10	197.10
AAMU	33-2000	2.01	+	199.18	181.10
1U	150-5000	2.37	-	181.10	138.10
13U	30-1500	2.47	+	197.16	169.12
1X	166-25000	2.42	+	167.10	110.10
3X	100-5000	2.41	-	164.90	122.00
37U	30-1000	2.38	+	197.10	182.10
7X	50-7500	2.39	+	167.00	124.10
1X*		2.40	+	174.10	115.10
QU*		2.38	-	188.10	143.10

Table S3. Matrix effect data for caffeine and its metabolites

Analyte	LQC		MQC		HQC	
	**Accuracy (%)**	**Precision (%)**	**Accuracy (%)**	**Precision (%)**	**Accuracy (%)**	**Precision (%)**
Caffeine	91.7	8.2	97.5	7.9	101.7	9.1
137U	76.6	8.0	90.1	9.7	95.8	2.2
13X	105.7	8.6	97.3	4.9	103.2	6.4
17X	89.5	13.4	100.3	2.4	91.7	1.0
37X	104.0	6.4	97.5	11.8	93.0	6.3
17U	97.4	11.3	96.2	2.7	103.2	3.7
AFMU	103.1	6.4	96.6	6.0	112.8	5.6
AAMU	68.2	8.6	81.1	6.9	85.3	6.4
1U	96.7	6.4	111.2	6.7	120.5	10.1
13U	100.3	6.4	104.0	2.5	113.2	2.5
1X	120.6	0.7	103.8	8.2	91.7	9.7
3X	97.3	1.0	97.4	8.7	101.7	1.3
37U	100.3	6.3	97.3	1.2	94.0	0.38
7X	103.2	3.7	100.3	3.4	102.2	4.5

Table S4. Recovery data for caffeine and its metabolites

Analyte	LQC		MQC		HQC	
	Accuracy (%)	Precision (%)	Accuracy (%)	Precision (%)	Accuracy (%)	Precision (%)
Caffeine	92.5	0.4	92.3	2.2	92.5	0.6
137U	96.8	10.5	95.4	4.7	98.3	2.6
13X	105.7	8.6	97.3	4.9	103.2	6.4
17X	109.2	2.1	88.3	4.8	109.1	9.4
37X	107.0	5.2	98.5	1.9	90.8	2.7
17U	105.8	8.2	92.5	0.4	104.4	1.4
AFMU	91.1	3.6	100.5	5.0	92.5	2.5
AAMU	92.5	3.6	95.5	3.6	92.5	3.6
1U	104.4	8.6	94.4	8.6	104.4	8.6
13U	103.2	2.1	113.2	2.1	103.2	4.9
1X	92.5	5.7	97.9	2.2	112.5	1.0
3X	109.1	5.2	99.1	5.2	109.1	4.8
37U	90.8	4.8	92.8	8.2	90.8	1.9
7X	112.5	1.9	102.5	5.7	112.5	0.4

Table S5. Stability of caffeine and its metabolites in various conditions

Analyte	Accuracy (%) ± CV (%)							
	Freeze and thaw stability		Processed sample stability		Short term stability		Long term stability	
	LOQ	HQ	LOQ	HQ	LOQ	HQ	LOQ	HQ
Caffeine	102.1 ± 2.5	103.5 ± 6.9	105.5 ± 0.9	110.1 ± 2.0	107.2 ± 1.2	98.3 ± 5.3	105.1 ± 2.6	101.5 ± 7.1
137U	101.4 ± 8.2	106.7 ± 3.3	105.8 ± 5.2	110.6 ± 3.3	110.8 ± 5.2	111.6 ± 3.3	100.8 ± 8.5	96.0 ± 10.5
13X	103.1 ± 11.4	100.9 ± 6.6	99.8 ± 3.8	98.9 ± 2.9	89.8 ± 3.8	88.9 ± 1.9	87.7 ± 2.1	90.1 ± 4.2
17X	100.9 ± 6.9	105.4 ± 8.8	101.9 ± 1.4	99.7 ± 8.2	110.9 ± 2.4	99.7 ± 8.2	100.9 ± 2.6	90.6 ± 3.8
37X	109.7 ± 3.7	110.7 ± 3.7	112.1 ± 2.8	110.3 ± 4.2	111.1 ± 2.8	111.3 ± 3.2	97.8 ± 7.1	105.8 ± 9.2
17U	100.3 ± 9.1	90.6 ± 5.4	95.2 ± 4.8	90.9 ± 5.1	91.2 ± 4.8	90.9 ± 5.1	89.4 ± 1.7	86.7 ± 0.9
AFMU	99.8 ± 9.4	90.6 ± 2.1	93.7 ± 2.7	88.9 ± 4.0	93.7 ± 2.7	89.9 ± 3.0	52.3 ± 1.4	47.2 ± 1.5
AAMU	102.1 ± 10.3	101.2 ± 6.3	111.9 ± 2.7	109.1 ± 5.7	111.9 ± 2.7	99.1 ± 9.7	305.2 ± 4.6	323.4 ± 10.8
1U	113.1 ± 0.9	106.4 ± 8.4	110.5 ± 2.8	91.6 ± 4.5	112.5 ± 2.8	91.6 ± 4.5	108.9 ± 4.2	107.4 ± 4.9
13U	111.2 ±1.5	100.6 ± 9.4	112.0 ± 4.8	107.9 ± 6.5	112.0 ± 0.8	108.9 ± 6.2	106.4 ± 4.4	108.0 ± 5.9
1X	100.5 ± 5.5	112.4 ± 0.7	108.0 ± 2.3	100.4 ± 2.3	108.0 ± 2.3	100.4 ± 8.8	108.0 ± 2.3	100.4 ± 10.1
3X	99.1 ± 2.0	99.9 ± 3.1	101.4 ± 6.5	105.0 ± 6.4	103.4 ± 6.5	105.0 ± 6.4	99.0 ± 12.4	111.2 ± 2.6
37U	107.8 ± 7.4	109.8 ± 2.4	100.9 ± 4.9	104.6 ± 2.7	108.1 ± 4.8	107.6 ± 4.4	94.8 ± 8.6	91.2 ± 4.3
7X	109.9 ± 5.4	108.8 ± 8.3	106.6 ± 0.8	109.1 ± 1.8	106.6 ± 0.8	109.1 ± 1.8	107.1 ± 5.8	101.5 ± 7.1
